# Identification and genetic characterization of a novel parvovirus associated with serum hepatitis in horses in China

**DOI:** 10.1038/s41426-018-0174-2

**Published:** 2018-10-23

**Authors:** Gang Lu, Lingshuang Sun, Jiajun Ou, Haibin Xu, Liyan Wu, Shoujun Li

**Affiliations:** 1 College of Veterinary Medicine, South China Agricultural University, Guangdong Province, 510642 Guangzhou, People’s Republic of China; 2 Guangdong Provincial Key Laboratory of Prevention and Control for Severe Clinical Animal Diseases, Guangdong Province, 510642 Guangzhou, People’s Republic of China; 3 Guangdong Technological Engineering Research Center for Pet, Guangdong Province, 510642 Guangzhou, People’s Republic of China

## Abstract

A novel equine parvovirus, equine parvovirus-hepatitis (EqPV-H), was first discovered in a horse that died of equine serum hepatitis in the USA in 2018. EqPV-H was shown to be a novel etiological agent associated with equine serum hepatitis. Following this initial report, no additional studies on EqPV-H have been published. In this study, a total of 143 serum samples were collected from racehorses at 5 separate farms in China and were analyzed to detect EqPV-H DNA via nested PCR. The results indicated a high prevalence of EqPV-H (11.9%, 17/143) in the studied animals. In addition, a remarkably high coinfection rate (58.8%, 10/17) with 2 equine flaviviruses (equine hepacivirus and equine pegivirus) was observed in the EqPV-H positive equines. However, all equines tested negative for Theiler’s disease-associated virus, an etiological agent associated with equine serum hepatitis. The genomes of six field EqPV-H strains were sequenced and analyzed, with the results indicating that the Chinese EqPV-H strains have low genetic diversity and high genetic similarity with the USA EqPV-H strain BCT-01. A phylogenetic analysis demonstrated that the Chinese EqPV-H strains clustered with BCT-01 in the genus *Copiparvovirus* but were distantly related to another equine parvovirus identified in horse cerebrospinal fluid. In addition, liver enzyme levels were detected in the EqPV-H positive serum samples, and all the values were in the normal range, indicating that infection can occur without concurrent liver disease. This study will promote an understanding of the geographical distribution, genetic diversity, and pathogenicity of EqPV-H.

## Introduction

As suggested by the International Committee on Taxonomy of Viruses (ICTV), the family *Parvoviridae* includes two subfamilies, *Densovirinae* and *Parvovirinae*. The latter contains eight genera: *Amdoparvovirus*, *Aveparvovirus*, *Bocaparvovirus*, *Copiparvovirus*, *Dependoparvovirus*, *Erythroparvovirus*, *Protoparvovirus*, and *Tetraparvovirus*. Until now, only two species had been officially included in the genus *Copiparvovirus*, *Ungulate copiparvovirus 1* (bovine parvovirus 2) and *Ungulate copiparvovirus 2* (porcine parvovirus 4)^1^. However, several newly identified parvovirus types have recently been classified in the genus *Copiparvovirus* based on sequence analyses, including sesavirus^2^, porcine parvovirus 5^3^, porcine parvovirus 6^4^, equine parvovirus-hepatitis (EqPV-H)^5^, and horse parvovirus cerebrospinal fluid^6^ ([CSF], which is provisionally designated EqPV-CSF in this study).

Equine serum hepatitis is also known as Theiler’s disease, equine acute hepatitis necrosis, serum sickness, and postvaccinal hepatitis. This disease was first described in 1919 by Arnold Theiler and is often associated with pretreatment with blood products in equines^7^. In 2013, a deep sequencing analysis of equine serum samples obtained during an equine serum hepatitis outbreak in the USA showed that a previously undescribed pegivirus of Theiler’s disease-associated virus (TDAV, also known as pegivirus D according to the ICTV) was the possible etiologic agent for this outbreak of equine serum hepatitis^8^. In 2018, Thomas J. Divers *et al* reported identifying a novel parvovirus of equine parvovirus-hepatitis (EqPV-H) in the serum and liver samples of a horse that died of equine serum hepatitis^5^. Although both viruses were grouped into the same genus, *Copiparvovirus*, a sequence analysis indicated that EqPV-H was genetically distinct from EqPV-CSF, another parvovirus identified in the CSF sample of a horse with neurological signs and lymphocytic pleocytosis^6^. Acute hepatitis was observed in horses after the administration of tetanus antitoxin containing EqPV-H. Unexpectedly, a retrospective study demonstrated that EqPV-H DNA was detected in all TDAV RNA-positive samples in the first report of TDAV. It is therefore possible that EqPV-H plays a major role in equine serum hepatitis.

Because EqPV-H is an emerging equine virus, data related to the geographical distribution, epidemiology, genetic diversity and clinical features of this viral infection are limited. Since its first identification, no further study on EqPV-H has been reported. In this study, we identified EqPV-H DNA in equines in Guangdong province in China and performed detailed genetic and clinical analyses.

## Results

### The prevalence of EqPV-H in equines in China

To increase our understanding of the infection and prevalence of EqPV-H in equines in China, a total of 143 equine serum samples were collected and tested. All the tested animals were racehorses and were an average age of 11.3 years old (range 1–21 years old), with most being geldings (115/143, 80.4%) and thoroughbreds (65/143, 45.5%) (Table 1).

**Table 1:** Information regarding the equine serum samples in our study

Farm	*n*	Sex^a^ M/F/G	Average age^b^ (range)	Breeds (n)	EqPV	EqHV	EPgV	TDAV
A	16	0/10/6	3.4(1–6)	Mixed breed (15); Akhal-teke horse (1)	2(12.5%)	3(18.8%)	0 (0%)	0(0%)
B	14	0/1/13	10(8–13)	Thoroughbred (14)	1(7.1%)	7(50.0%)	0 (0%)	0(0%)
C	29	1/0/28	10.1(6–18)	Thoroughbred (22); Warmblood (7)	7(24.1%)	5(17.2%)	1(3.4%)	0(0%)
D	30	2/6/22	13.8(3–21)	Thoroughbred (7); Warmblood (14); Pony (6); Arabian horse (3)	3(10%)	6(20.0%)	2(6.7%)	0(0%)
E	54	2/6/46	13.1(2–20)	Thoroughbred (22); Warmblood (16); Pony (15); Mixed breed (1)	4(7.4%)	11(20.4%)	0 (0%)	0(0%)
Total	143	5/23/115	11.3(1–21)	Thoroughbred (65); Warmblood (37); Pony (21); Mixed breed (16); Arabian horse (3); Akhal-teke horse (1)	17(11.9%)	32(22.4%)	3(2.1%)	0(0%)

^a^M/F/G: male/female/gelding

^b^Age in years

Serum EqPV-H DNA was detected by two independent nested-PCR procedures targeting the partial NS and VP genes. After electrophoresis, a total of 17 equine serum samples presented a band with the expected size of the partial VP and/or NS gene. The subsequent sequencing and BLAST results from the NCBI database demonstrated that all of the sequences best matched the EqPV-H strain BCT-01 (nucleotide homology, >98%) when both the partial NS and VP genes were analyzed. The results indicated that of 17 equine serum samples assayed in this study contained EqPV-H DNA, a prevalence of 11.9% (Table 1). It was noted that the prevalence of EqPV-H varied in different collection locations and ranged from 7.1% to 24.1%.

The RNA of three newly discovered flaviviruses (equine hepacivirus [EqHV, also known as hepacivirus A according to the ICTV]^9,10^, equine pegivirus [EPgV, also known as pegivirus E according to the ICTV]^11^, and TDAV) were also identified in the samples. These results demonstrated that EqHV and EPgV circulate in the equine population with prevalences of 22.4% (32/143), and 2.1% (3/143), respectively (Table 1). A remarkably high prevalence of 50.0% (7/14) was observed for EqHV RNA in farm B. However, no TDAV RNA was identified in any of the tested equines at this site.

Detailed information on the equine samples testing positive for EqPV-H DNA is listed in Table 2. Most of the EqPV-H viremia animals were geldings (15/17) and thoroughbreds (9/17), with ages ranging from 3 to 21 years old. Nine and 3 EqPV-H viremia animals were observed to be coinfected with EqHV and EPgV, respectively, whereas two animals (C24 and D4) from separate farms were coinfected with all three viruses (EqPV-H, EqHV, and EPgV).

**Table 2:** Information regarding the equine serum samples containing EqPV-H DNA in our study

Farm	Equine ID	Sex^a^ M/F/G	Age^b^	Breed	Co-infection^c^	
					EqHV	EPgV
A	A3^d^	G	3	Mixed breed		
	A11	F	4	Mixed breed		
B	B14	G	12	Thoroughbred	+	
C	C3	G	18	Thoroughbred		
	C4	G	8	Warmblood	+	
	C11^d^	G	12	Warmblood	+	
	C14^d^	G	17	Thoroughbred		
	C16	G	10	Thoroughbred		
	C22	G	10	Thoroughbred	+	
	C24	G	7	Thoroughbred	+	+
D	D4^d^	G	21	Thoroughbred	+	+
	D9	G	13	Warmblood		
	D29	M	12	Warmblood		+
E	E15	G	11	Thoroughbred	+	
	E35^d^	G	10	Pony		
	E36^d^	G	19	Pony	+	
	E38	G	12	Thoroughbred	+	

^a^M/F/G: male/female/gelding

^b^Age in years

^c^Co-infection indicated by “+”

^d^The EqPV-H genome was sequenced

### Sequence analysis

The complete coding sequences of six field EqPV-H strains were acquired from samples obtained at Farms A, C, D, and E (Supplementary Fig. S1). Due to the limited sample amount and low viral content in sample B14, the genome of the EqPV-H strain identified at Farm B was not further studied. The six EqPV-H strains sequenced in this study were named A3, C11, C14, D4, E35, and E36 according to the equine ID of their host. These genome sequences have been submitted to the GenBank database with the accession numbers MH500787-MH500792.

The genome of the first reported EqPV-H strain, BCT-01, is 5,308 nucleotides in length and contains two predicted coding regions for the NS (1,779 nucleotides) and VP (2,922 nucleotides) proteins, each of which is followed by intergenic regions of 21 (intergenic region 1) and 583 (intergenic region 2) nucleotides, respectively^5^. The genomes of all six field EqPV-H strains presented the same NS, VP, and intergenic region 1 nucleotide lengths as BCT-01, as well as equal G + C content for the NS (53.48–53.70%) and VP (47.01–47.52%) coding regions as BCT-01 (NS, 53.37%; VP, 47.49%). In addition, the nucleotide sequence of intergenic region 1 (TGAAACGTAAGTTTTCACACC) was strictly conserved among all the EqPV-H strains, including BCT-01. For intergenic region 2, because only a partial 18-nucleotide sequence was obtained by PCR, it was not further investigated.

The NS and VP nucleotide and protein sequences of the field EqPV-H strains were compared to those of BCT-01 and other viruses in the genus *Copiparvovirus* and sequence homologies were estimated. Compared with BCT-01, a total of six (A171G, A504C, A563C, G590A, T1221C, and C1778A) and eighteen (G267T, C685A, G872A, C899A, A973G, T978C, G981A, G982A, G983A, C1662T, G1881C, T2103C, G2136A, T2190G, T2271A, C2685T, G2730A, and C2901A) unique nucleotide substitutions were observed in the NS and VP genes, respectively, in all the Chinese EqPV-H strains (Supplementary Fig. S2, 3). These substitutions caused three unique amino acid substitutions each in NS (K188T, S197N, and P593H) and VE (G291D, A328K, and H730Q). The NS gene identified in the Chinese EqPV-H strains shared nucleotide similarities of 97.2-98.9%, 43.0-43.4%, and 41.2–46.1% and amino acid similarities of 98.1–99.2%, 29.3–29.5%, and 27.3–34.8% with BCT-01, EqPV-CSF, and other copiparvoviruses, respectively (Table 3). In addition, the NS-coding sequence of the Chinese EqPV-H strains shared nucleotide and amino acid similarities of 97.0–99.7% and 98.1–99.7% with each other, respectively. The VP-coding sequence of the Chinese EqPV-H strains had nucleotide similarities of 96.8–98.2%, 37.3–37.7%, and 39.2–46.5% and amino acid similarities of 96.8–97.5%, 27.7–28.2%, and 27.8–35.0% with BCT-01, EqPV-CSF, and other copiparvoviruses, respectively (Table 4). Among the six field-obtained Chinese EqPV-H strains, the level of nucleotide and amino acid similarity was 96.7–99.3% and 96.0–99.3%, respectively.

**Table 3:** Nucleotide (upper right) and amino acid (bottom left) similarity of the NS coding sequence between the Chinese EqPV-H strains and other members of the genus *Copiparvovirus*

	BPV2	Sesavirus	PPV4	PPV5	PPV6	EqPV-CSF	BCT-01	A3	C11	C14	D4	E35	E36
BPV2		45.2	48.6	50.0	45.0	51.2	45.8	45.6	45.8	45.7	45.9	46.1	46.0
Sesavirus	27.7		41.9	41.5	41.1	44.2	41.3	41.5	41.2	41.2	41.5	41.3	41.3
PPV4	36.6	27.5		78.5	58.2	44.2	45.5	46.0	45.7	45.8	45.8	46.0	46.0
PPV5	36.0	28.0	85.4		57.5	47.0	45.2	45.7	45.1	45.2	45.5	45.8	45.7
PPV6	34.1	28.0	59.0	57.7		40.8	42.5	42.7	43.1	43.2	42.7	42.9	42.7
EqPV-CSF	30.1	28.2	31.1	31.9	29.3		43.1	43.4	43.0	43.0	43.2	43.4	43.4
BCT-01	32.8	27.5	33.6	34.4	33.6	29.3		98.4	97.2	97.5	98.9	98.8	98.7
A3	32.6	27.3	33.8	34.8	33.6	29.5	99.2		97.0	97.4	98.7	98.9	98.8
C11	32.8	27.5	34.0	34.4	33.3	29.5	98.1	98.3		99.2	97.1	97.3	97.1
C14	33.0	27.5	33.8	34.4	33.3	29.3	98.5	98.7	99.7		97.8	97.8	97.6
D4	32.8	27.3	33.6	34.4	33.3	29.3	99.2	99.3	98.3	98.7		98.9	98.8
E35	32.8	27.5	34.0	34.8	33.6	29.5	98.7	99.2	98.1	98.5	98.8		99.7
E36	32.8	27.5	33.8	34.6	33.4	29.3	99.0	99.5	98.1	98.5	99.2	99.7	

*BPV* bovine parvovirus, *PPV* porcine parvovirus

**Table 4:** Nucleotide (upper right) and amino acid (bottom left) similarity of the VP coding sequence between the Chinese EqPV-H strains and other members of the genus *Copiparvovirus*

	BPV2	Sesavirus	PPV4	PPV5	PPV6	EqPV-CSF	BCT-01	A3	C11	C14	D4	E35	E36
BPV2		45.9	44.2	42.3	39.0	46.2	39.4	39.2	39.2	39.2	39.4	39.2	39.2
Sesavirus	25.5		45.2	42.8	43.7	43.9	41.4	41.4	41.3	41.4	41.5	41.1	41.0
PPV4	32.8	37.4		61.1	48.1	42.6	46.2	46.1	46.0	46.4	46.4	46.5	46.5
PPV5	29.1	30.2	54.4		45.0	39.1	45.2	45.2	45.5	45.5	45.4	45.4	45.5
PPV6	26.4	31.4	42.8	37.4		36.8	40.9	40.3	40.5	40.6	40.2	40.9	40.8
EqPV-CSF	28.5	28.9	33.9	29.4	25.9		37.4	37.4	37.3	37.5	37.7	37.6	37.5
BCT-01	28.3	32.4	34.8	30.4	30.5	27.9		98.2	97.6	97.7	98.0	96.8	97.0
A3	28.3	32.0	34.5	30.3	30.4	28.2	97.5		97.5	97.8	98.6	96.7	96.9
C11	28.1	32.5	34.8	30.5	30.3	28.0	96.8	96.3		98.4	97.4	97.1	97.1
C14	27.9	32.4	34.7	30.3	30.4	27.8	97.1	96.7	96.4		98.9	97.4	97.5
D4	27.9	32.3	34.7	30.4	30.3	27.7	97.1	97.4	96.0	99.3		97.0	97.1
E35	27.8	32.4	35.0	30.1	30.1	27.8	97.2	96.5	96.5	96.8	96.8		99.3
E36	27.9	32.4	34.8	30.3	30.3	27.8	97.5	96.8	96.2	96.9	96.9	98.5	

*BPV* bovine parvovirus, *PPV* porcine parvovirus

### Phylogenetic analysis

**Fig. 1:**
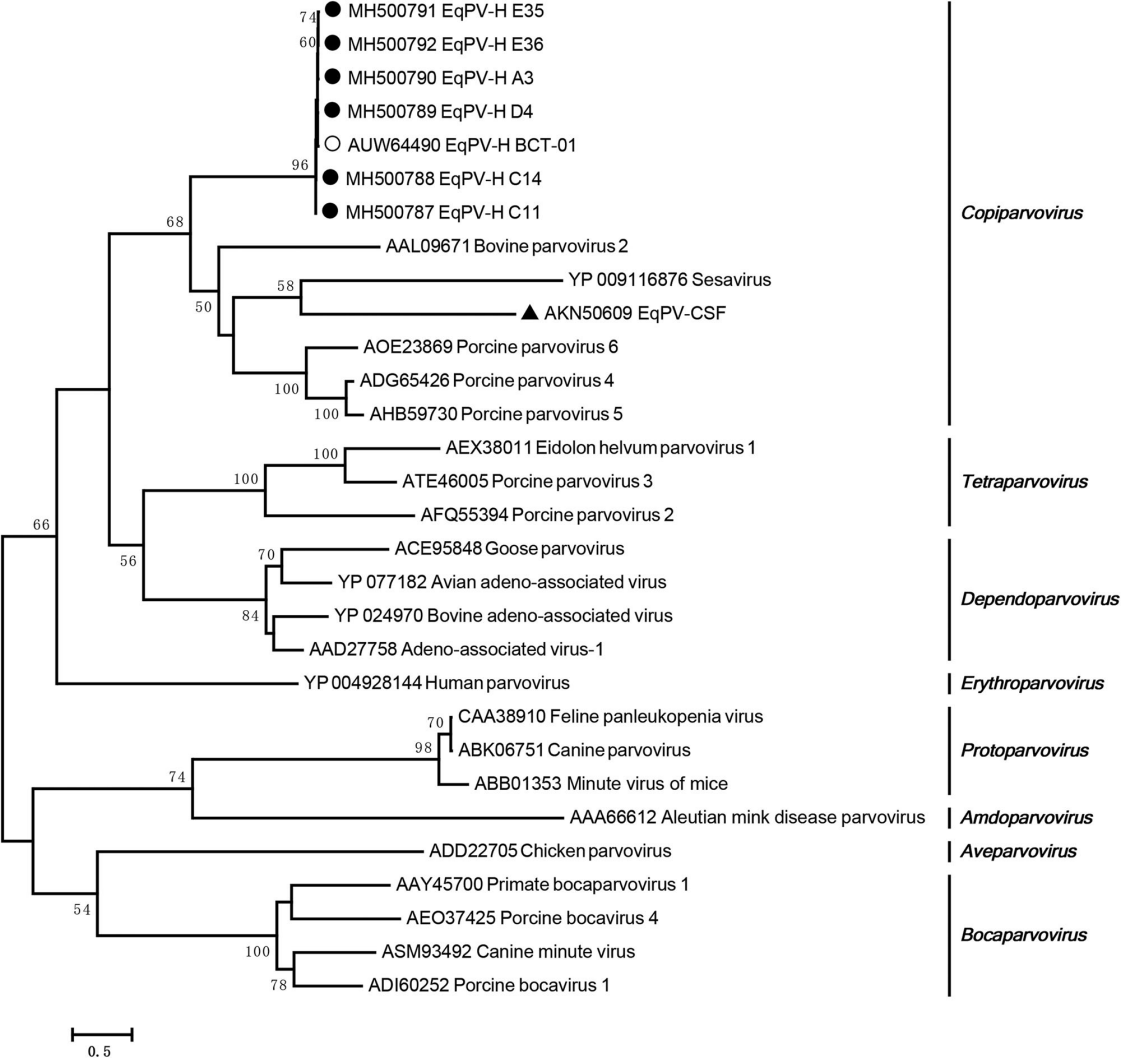
Phylogenetic analysis of Chinese EqPV-H strains based on the NS protein. The Chinese EqPV-H strains, BCT-01, and EqPV-CSF are indicated by a fixed circle, an open circle, and a triangle, respectively

The EqPV-H NS protein is used to perform phylogenetic analyses because it is more conserved than the VP protein^5^. A phylogenetic analysis demonstrated that all the Chinese EqPV-H strains had a close relationship with BCT-01 and that they were clustered together with bovine parvovirus, porcine parvovirus 4, porcine parvovirus 5, and porcine parvovirus 6 in the genus *Copiparvovirus* (Fig. 1). As previously reported, EqPV-H strains are distantly related to another equine parvovirus, EqPV-CSF, which clusters with sesavirus.

### Clinical examination

The activities of liver-specific enzymes (AST [aspartate aminotransferase], γ-GT [γ-glutamyltransferase] and GLDH [glutamate dehydrogenase]) in the EqPV-H-positive serum samples were measured and were within the reference ranges (Supplementary Fig. S4), with no evidence of liver disease in the equines observed.

## Discussion

In this study, the presence of a novel equine parvovirus, EqPV-H, associated with serum hepatitis was observed for the first time in equines in China. The average prevalence of EqPV-H viremia was 11.9% (17/143) by PCR. At an equine farm, approximately 25% of the tested equine serum samples contained EqPV-H DNA (Table 1). In the first report of EqPV-H in the USA, a prevalence of 13.0% (13/100) was described by PCR^3^. In addition, an antibody specific for an EqPV-H VP antigen was identified in two EqPV-H DNA-negative equines, indicating a possible EqPV-H clearance event. However, the detection limit of PCR may have influenced the detection of EqPV-H DNA in the tested animals. The investigations in China and the USA suggest a high frequency of EqPV-H infection in equines. The EqPV-H prevalence and infection rates in the equine population in other countries remain to be determined.

Previous studies demonstrated that parvovirus in humans and other animals can be transmitted via multiple routes, including plasma-derived products^12^, the fecal-oral route^13^, and the respiratory tract^14^. EqPV-H DNA-negative equines administered a contaminated tetanus antitoxin were infected with EqPV-H, suggesting that this virus can be transmitted via equine blood products^5^. However, the equines tested in this study were not treated with any equine blood products, as confirmed by the equine owner. Whether EqPV-H can be transmitted via other routes still needs to be further studied.

In addition to EqPV-H DNA, EqHV, EPgV, and TDAV RNA were also tested to investigate whether equines can be coinfected with these viruses (Tables 1, 2). Consistent with our previous results^15^, the prevalence of EqHV (22.4%) was markedly higher than those of EPgV (2.1%) and TDAV (0.0%). In addition, a high frequency of coinfection with EqHV (9/17, 52.9%) was observed in equines with EqPV-H viremia. It is interesting that although only 3 equines had EPgV viremia in our study, all of the serum samples obtained from these animals contained EqPV-H DNA. Similarly, human parvovirus 4 infection is common in hepatitis C virus-infected individuals, up to 31% in a previous report^16^. In addition, a viral metagenomics analysis of 51 plasma pools obtained from 498 individuals indicated a high prevalence (26/38, 68.4%) of human pegivirus RNA in human parvovirus B19 DNA-positive samples^17^. A previous study performed in the USA indicated that EqPV-H has a low genetic diversity (<2%) in both the NS and VP genes^5^. Consistent with this finding, we sequenced the genomes of six field EqPV-H strains and observed a very low genetic diversity in the NS and VP genes (<3% and <4%, respectively) (Tables 3, 4). However, field EqPV-H strains exhibited a distinct geographical pattern. Unique nucleotide substitutions were identified in the strains obtained from the same farm. For example, both strains C11 and C14 were observed at Farm C, and a total of 28 unique nucleotide substitutions were identified in their NS genes that were not present in BCT-01, A3, D4, E35, or E36.

As a newly discovered equine virus, the relationship between EqHV infection and equine liver disease continues to be debated^18–22^. Although EPgV was first identified in horses with elevated liver enzyme levels, EPgV is not believed to be associated with equine hepatopathy^11,20^. Animal experiments have demonstrated that EqPV-H can increase liver enzyme levels and cause histopathological changes. However, none of the 13 naturally infected equines tested showed biochemical evidence of liver disease in the first report of EqPV-H^5^. In this study, among the 17 EqPV-H-positive equines, 10 animals were coinfected with either EqHV or EPgV, and 2 animals were coinfected with all three viruses. However, the liver enzyme levels of all the animals were within the normal range. This further supports previous findings showing that infection can occur without concurrent liver disease^5^.

In conclusion, this is the first report of a high prevalence of EqPV-H in equines in China, with the identified EqPV-H strains having low genetic diversity. Further investigations are needed to understand the worldwide epidemiology of EqPV-H and its genetic diversity and pathogenicity.

## Materials and methods

### Sample collection

In 2018, a total of 143 equine serum samples were collected from racehorses at five equine farms in Southern China. The detailed information of the samples is listed in Table 1. The samples were acquired with the permission of the owners of the animals. All serum samples were stored at −70 °C immediately after collection until further testing.

### Virus detection

The presence of viral RNA/DNA of three emerging equine flaviviruses (EqHV, EPgV, and TDAV) and EqPV-H was analyzed in equine serum by RT-PCR/PCR. Briefly, total viral RNA/DNA was extracted from 400 µL of serum obtained from each of the 120 samples using a TaKaRa minibest viral RNA/DNA extraction kit (Takara, China). For each sample, 8 µL of viral RNA was subsequently used for cDNA synthesis. EqHV, EPgV, and TDAV RNA were detected using the cDNA as a PCR template according to the experimental procedure described in our previous studies^15,23,24^. EqPV-H DNA was detected by nested PCR using Genestar taq polymerase premix (Kangrun, China) according to the manufacturer’s instructions with the PCR primers reported by Thomas J. Divers et al.^5^. PCR products were detected by 1% gel electrophoresis and visualized under UV light. The PCR products with the expected size were purified by gel extraction using a Tiangen universal DNA purification kit (Tiangen, China) and were sequenced from both ends (BGI, China). Finally, the raw sequences were used to perform BLAST analyses (https://blast.ncbi.nlm.nih.gov/Blast.cgi) to assess the presence of the detected virus.

### Viral genome sequencing and analysis

To obtain the viral genome of the prevalent EqPV-H strains in Chinese equines, two PCR primer pairs (1104F [ATGGAGACCTTTTGGTACGG] and 1104R [GGGAATGTCATTGAACGGGAA]; and 1690F [TCAAACACGTCGCTGCATTC] and 1690R [CAACACGATTTTATTGCATTACCGT]) were designed using Oligo 7.0. according to the sequence of the first reported EqPV-H strain, BCT-01 (GenBank accession no. MG136722). The primers flanked the partial nucleotide coding sequences of the NS and VP proteins, and PCR was performed using Phanta max superfidelity DNA polymerase (Vazyme, China). After gel electrophoresis and DNA purification, the DNA fragments were cloned into pCloneEZ-blunt (Clone smarter, USA) and then transformed into *E. coli* DH5α competent cells. The *E. coli* clones were picked and evaluated, and positive clones were subsequently sequenced. The raw sequence data were further assembled and processed using SeqMan 7.1.0. Based on the sequencing results, another primer pair (2508F [AGACGGGGAAACGTAATGCT] and 2508R [CTACCACACCGACAGTTGTA/G]) containing degenerate bases was used for gap-filling PCR to obtain the complete nucleotide coding sequences of the NS and VP proteins.

The nucleotide and protein sequences of the field EqPV-H strains obtained in this study, EqPV-CSF, and other representative viral strains in the genus *Copiparvovirus* were aligned with those of BCT-01 using BioEdit 7.0.9.0. using Clustal W. The nucleotide and protein similarities between these strains were calculated using MegAlign 7.1.0, the results of which are displayed in Tables 3, 4.

### Phylogenetic analysis

To understand the relationship between the prevalent EqPV-H strains identified in Chinese equines in this study and other *Parvovirinae* members, representative viral strains were retrieved from the GenBank database. A maximum likelihood phylogenetic tree based on the NS protein amino acid sequences was generated with MEGA 5.05 using rtREV with Freqs. (+F), gamma distribution with invariant sites (G+I) substitution models and a bootstrapping value of 1,000 (Fig. 1).

### Liver enzyme detection

Biochemical tests for AST, γ-GT, and GLDH levels in each of the 17 EqPV-H DNA-positive equine serum samples were performed using ELISA kits (Lapai, China) according to the manufacturer’s instructions. Enzyme levels were considered elevated if they exceeded the upper limit of the normal range (AST, 582 ng/L; γ-GT, 965 ng/L; and GLDH, 796 ng/L). The ranges were determined by >100 serum samples collected from healthy equines. Briefly, a reference standard with known equine AST, γ-GT, and GLDH contents was diluted in a specific proportion, and standard curves were generated based on the OD_450_ absorbance value of the reference standard. The liver enzymes in the equine samples were calculated according to the standard curves.

## Electronic supplementary material

Supplementary Fig.S1

Supplementary Fig.S2

Supplementary Fig.S3

Supplementary Fig.S4
