# Supplementary figures and images for: Identification and genetic characterization of a novel parvovirus associated with serum hepatitis in horses in China
Source: Emerg Microbes Infect. 2018 Oct 23;7:170. doi: 10.1038/s41426-018-0174-2 (PMC6198012; doi:10.1038/s41426-018-0174-2)

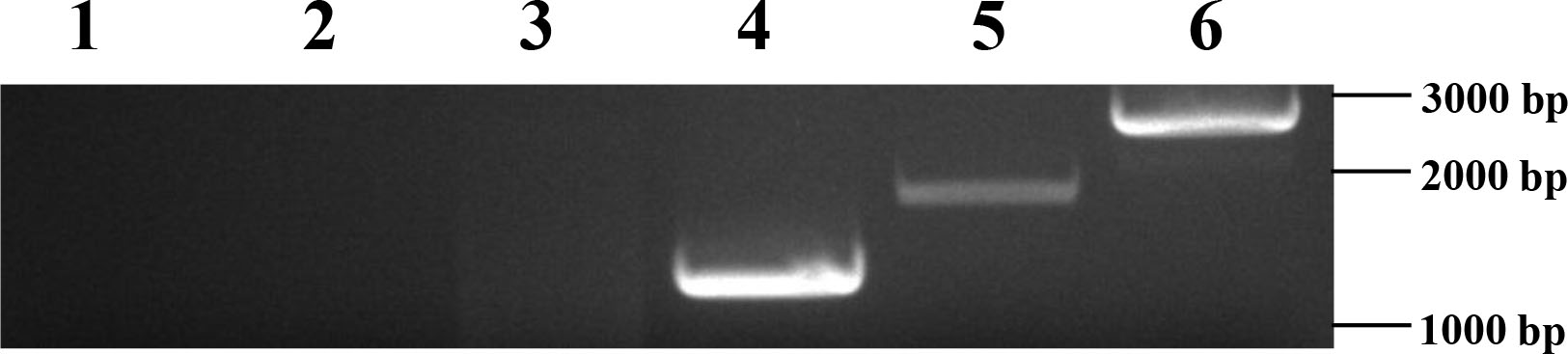

Supplement: Supplementary file 1 — Supplementary Fig.S1 [file 41426_2018_174_MOESM1_ESM.jpg]

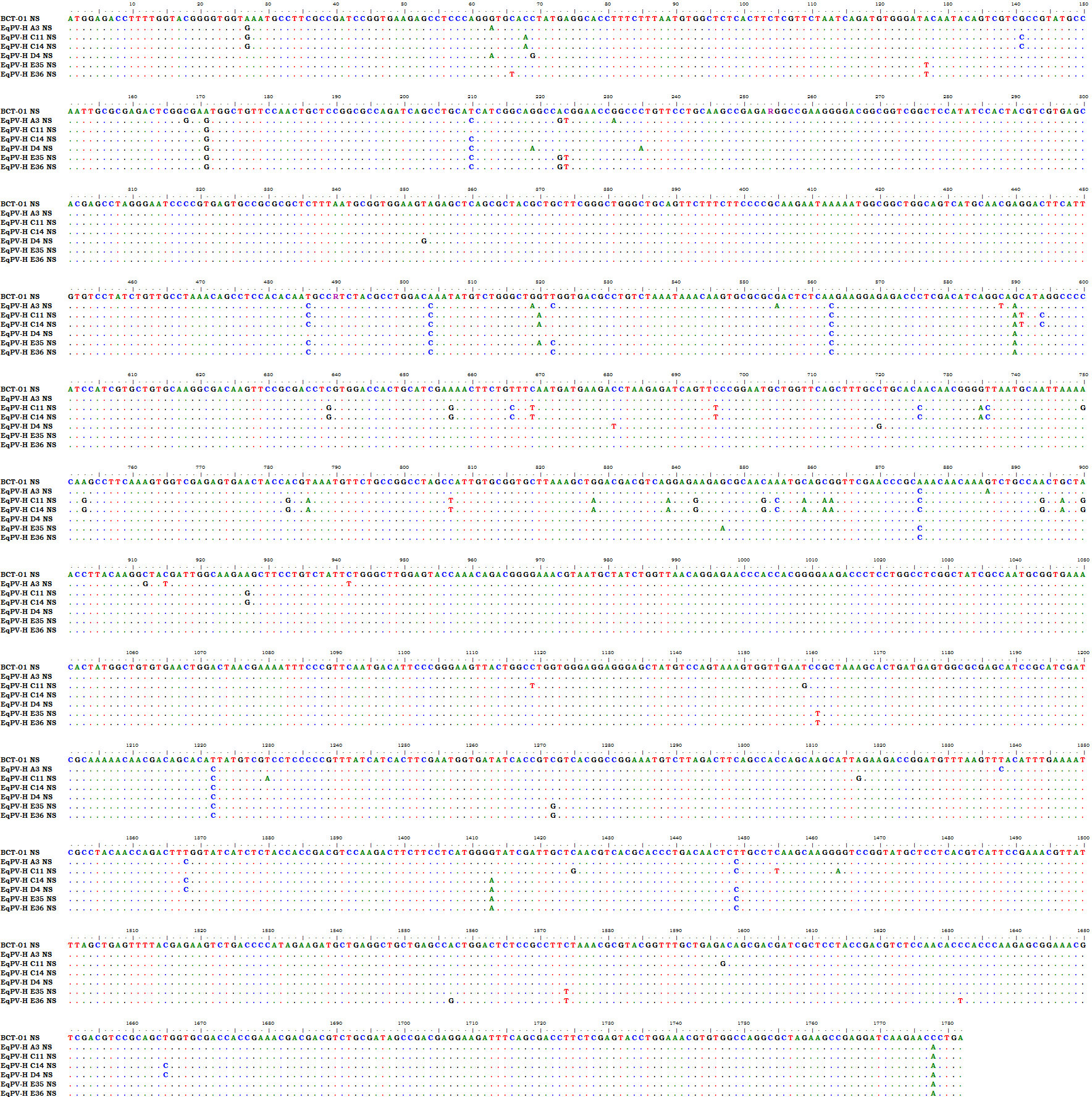

Supplement: Supplementary file 2 — Supplementary Fig.S2 [file 41426_2018_174_MOESM2_ESM.jpg]

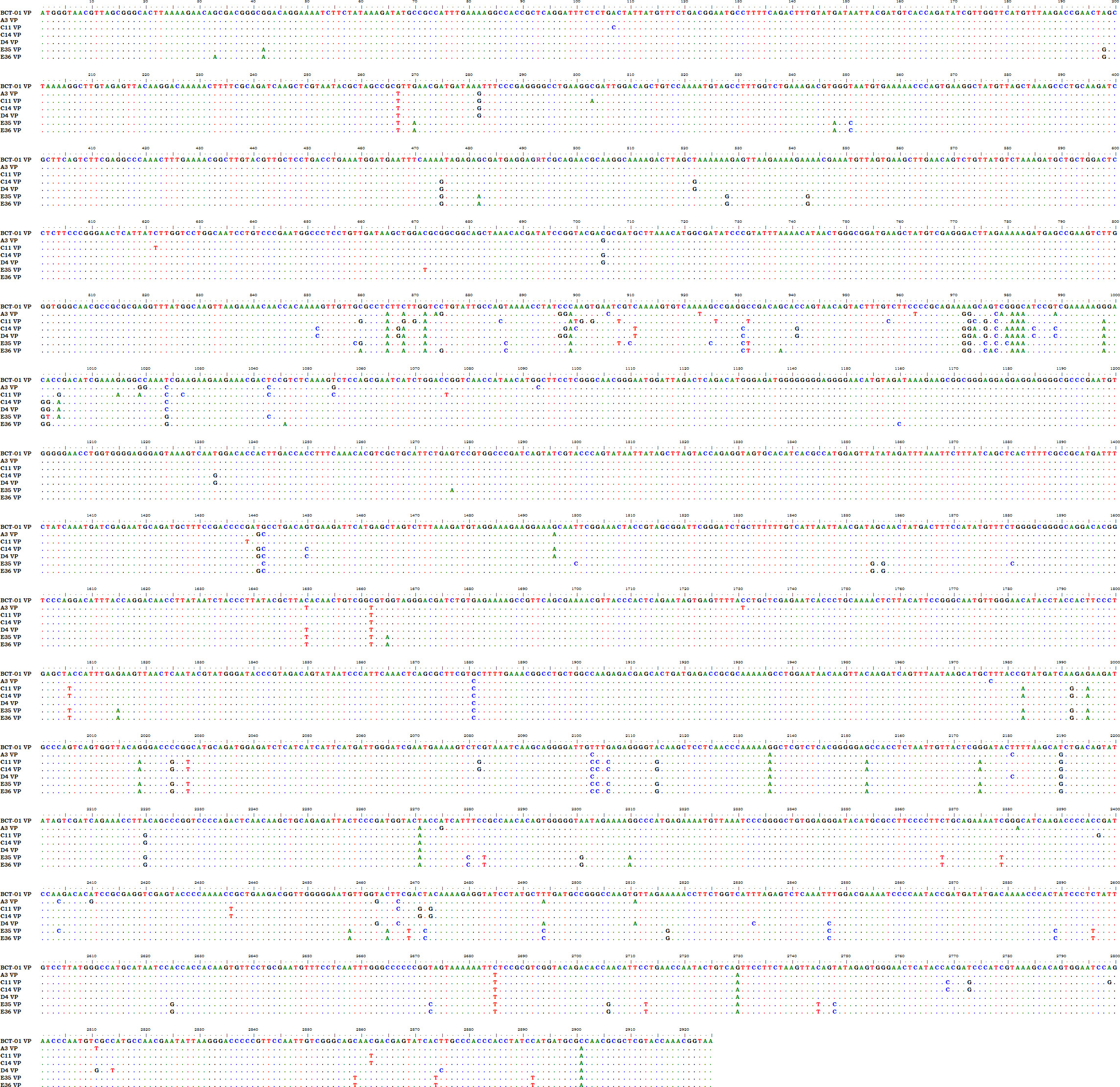

Supplement: Supplementary file 3 — Supplementary Fig.S3 [file 41426_2018_174_MOESM3_ESM.jpg]

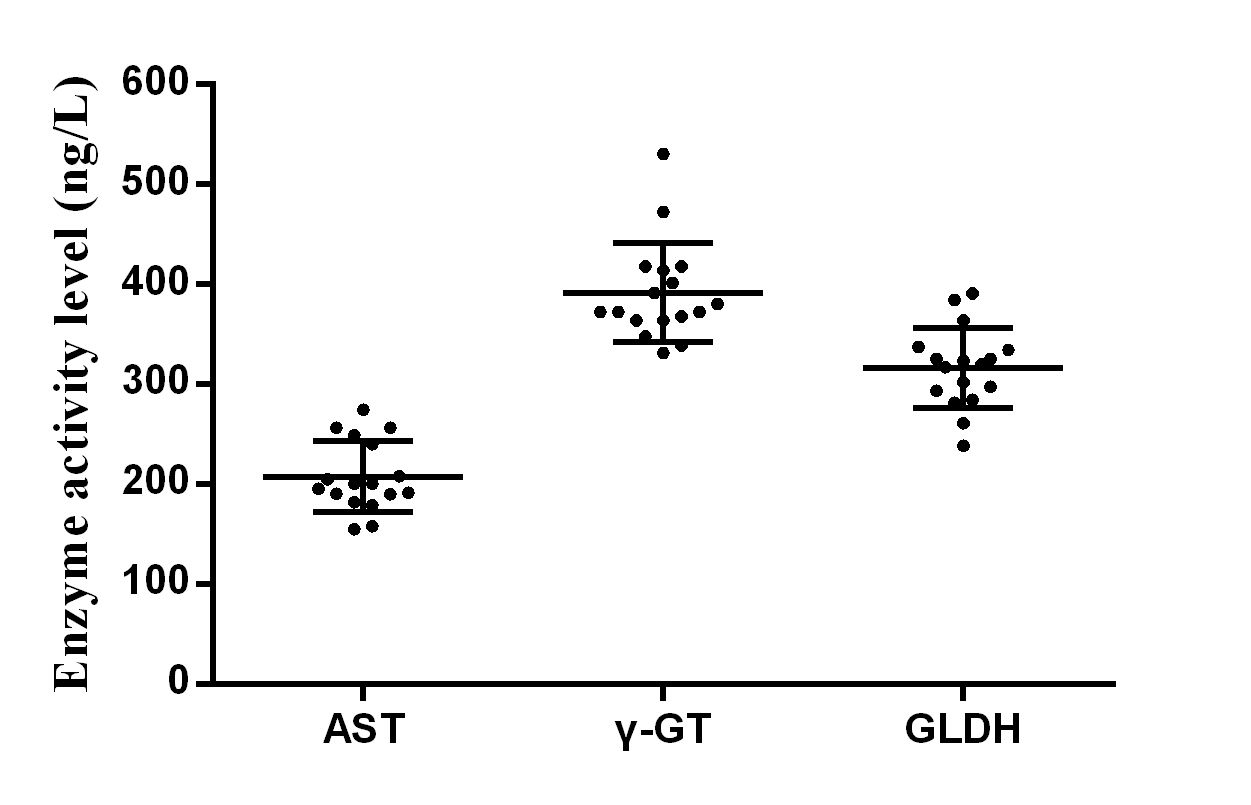

Supplement: Supplementary file 4 — Supplementary Fig.S4 [file 41426_2018_174_MOESM4_ESM.jpg]
